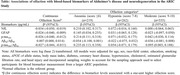# Olfaction and blood‐based biomarkers of Alzheimer’s disease pathology and neurodegeneration in the ARIC Study

**DOI:** 10.1002/alz.088361

**Published:** 2025-01-09

**Authors:** Srishti Shrestha, Xiaoqian Zhu, Kevin J. Sullivan, Priya Palta, Jennifer A. Deal, Andrea L.C. Schneider, Honglei Chen, Rebecca F. Gottesman, Bharat Thyagarajan, B Gwen Windham, Thomas H. Mosley, Michael E. Griswold, Vidyulata Kamath

**Affiliations:** ^1^ University of Mississippi Medical Center, The MIND Center, Jackson, MS USA; ^2^ University of North Carolina Chapel Hill, Chapel Hill, NC USA; ^3^ Johns Hopkins Bloomberg School of Public Health, Baltimore, MD USA; ^4^ University of Pennsylvania Perelman School of Medicine, Philadelphia, PA USA; ^5^ Michigan State University, East Lansing, MI USA; ^6^ National Institute of Neurological Disorders & Stroke Intramural Research Program, National Institute of Health, Bethesda, MD USA; ^7^ University of Minnesota, Minneapolis, MN USA; ^8^ University of Mississippi Medical Center, Jackson, MS USA; ^9^ Johns Hopkins University School of Medicine, Baltimore, MD USA

## Abstract

**Background:**

Olfactory impairment appears early in the course of Alzheimer’s Disease (AD) and may serve as a non‐invasive early marker of AD. Few studies have examined the association between olfaction and blood biomarkers of AD neuropathology in large, diverse, community‐based populations. Blood levels of amyloid‐beta (Aβ_42_ and Aβ_40_), phosphorylated‐tau (p‐tau) forms, glial fibrillary acidic protein (GFAP), and neurofilament‐light chain (NfL) appear to reliably reflect corresponding brain neuropathologies and neurodegeneration. Investigation of olfaction in relation to these biomarkers could provide insights into its utility in the early identification of individuals at high risk of progressing to AD.

**Methods:**

We investigated cross‐sectional associations of olfaction (measured by a 12‐item odor identification test) with blood Aβ_42_/Aβ_40_ ratio, GFAP, NfL, p‐tau_181_, and p‐tau_181_/Aβ_42_ ratio (measured using Quanterix ultrasensitive single‐molecule array assays) in 1,565 Black and White participants from the community‐based ARIC Study and whether these associations differed by race and APOE ε4 genotype. Both olfaction and blood biomarkers were obtained at ARIC visit 5 (2011‐2013). Separate linear regression models were used to examine the association of continuous olfaction score and olfaction categories (anosmia: score ≤6; hyposmia: score 7‐8; moderate olfaction: score 9‐10, normal olfaction: score 11‐12) with each biomarker (all biomarkers were log‐transformed and measured in pg/mL), adjusting for relevant covariates (See Table).

**Results:**

Among 1,565 participants (mean ± SD age:76±5 years, 60% women, 27% self‐reported Black), the mean ± SD olfaction score was 9.2±2.3; 14% had anosmia. Consistent with our hypotheses, higher olfaction score (i.e., better olfactory function) was significantly associated with lower levels of NfL, GFAP, p‐tau_181_, and p‐tau_181_/Aβ_42_, and higher Aβ_42_/Aβ_40_ ratio (See Table). Compared to normal olfaction, anosmia (i.e., poor olfaction) was significantly associated with higher NfL, GFAP, p‐tau_181_, and p‐tau_181_/Aβ_42_ ratio and lower (although not statistically significant) Aβ_42_/Aβ_40_ ratio. Associations of hyposmia and moderate olfaction with these biomarkers were not statistically significant. The associations did not differ by APOE ε4 status or race.

**Conclusion:**

Our findings suggest that poor olfaction is associated with concurrent blood biomarkers of AD and neurodegeneration. Future analyses will examine associations of olfaction with prospective changes in these biomarkers and if these associations are explained by relevant neuroimaging markers.